# Comparison of Interferon-Gamma Release Assay and Tuberculin Skin Test for the Screening of Latent Tuberculosis in Inflammatory Bowel Disease Patients: Indian Scenario

**DOI:** 10.1155/2021/6682840

**Published:** 2021-01-26

**Authors:** Alok Kumar Mantri, Priti Meena, Amarender Singh Puri, Ajay Kumar, Sanjeev Sachdeva, Siddharth Srivastava, K. Arivarasan, Shivakumar Varakanahali

**Affiliations:** ^1^Department of Gastroenterology, G. B. Pant Institute of Postgraduate Medical Education and Research (GIPMER), New Delhi, India; ^2^Institute of Renal Science, Sir Gangaram Hospital, New Delhi, India; ^3^Kauvery Hospital Trichy, Tennur, India; ^4^Narayana Multispeciality Hospital, HSR Layout, Bangalore, India

## Abstract

**Background:**

In a country like India, where the prevalence of tuberculosis is very high, the role of screening tools for detection of latent tuberculosis infection (LTBI) like TST and IGRA is still unclear, especially in inflammatory bowel disease (IBD) patients. Our study is aimed at comparing the interferon-gamma release assay (IGRA) and tuberculin skin test (TST) to determine the prevalence of LTBI in IBD patients in the Indian subset of the population.

**Methods:**

It was a prospective observational analysis. A total of 257 participants were included in the study. Both TST and IGRA were performed in consecutive patients diagnosed with IBD (131 patients) and in 126 healthy individuals. Both tests were performed on the same day. LTBI diagnosis was considered if any one of TST or IGRA was found to be positive.

**Results:**

Out of 131 IBD patients, 121 patients had ulcerative colitis and 10 patients had Crohn's disease. 29% of the IBD patients and 22% of the control subjects had LTBI. The study demonstrated concordance between TST and IGRA. Agreement test kappa value for IBD patients was 0.656 (CI 0.50-0.81), with a *p* value of <0.001, suggestive of a fair agreement. Mean IFN-*γ* release was lower in the immunosuppressed group as compared to non-immunosuppressed individuals (0.26 ± 0.17 vs. 0.45 ± 0.07, *p* = 0.02). Cohen's kappa coefficient values in IBD cases and control subjects were 0.66 and 0.79, respectively. TST was found to be negatively correlated to BMI.

**Conclusion:**

Agreement between TST and IGRA was fair in IBD patients. For LTBI screening in IBD patients, TST and IGRA are complementary methods.

## 1. Introduction

Inflammatory bowel disease (IBD) currently represents a substantial economic burden affecting nearly 4 million people worldwide [1]. Crohn's disease (CD) and ulcerative colitis (UC) are the most common idiopathic IBD [2]. The mainstay of treatment includes anti-inflammatory agents, immunosuppressive drugs, and antitumor necrosis factor (anti-TNF) therapy [3]. While biologicals have been shown to improve prognosis, increased utilization and earlier administration of these therapies have led to a growing concern about the risk of opportunistic infections. Of particular concern is the potential reactivation of latent tuberculosis infection (LTBI), which in this clinical scenario often presents in an aggressive and disseminated fashion. LTBI is defined as a state of persistent bacterial viability, immune control, and no evidence of clinically manifested active tuberculosis (TB) [4, 5]. Currently, it is not possible to directly diagnose LTBI in humans. So, the diagnosis of LTBI is based on information gathered from the medical history, tuberculin skin test (TST) or interferon-gamma release assay (IGRA) result, chest radiograph, physical examination, and in certain circumstances, sputum examinations. Although several studies assessed performances of IGRA and TST in western populations, there is very limited data in populations with high background rates of TB and bacille Calmette-Guerin (BCG) vaccination. In a country like India, where the prevalence per lakh for tuberculosis is as high as 230 and the prevalence of LTBI is nearly 50%, the role of TST and IGRA is still unclear, especially in IBD patients [6, 7]. Our study is aimed at comparing the interferon-gamma release assay and tuberculin skin test to determine the prevalence of latent tuberculosis in inflammatory bowel disease patients in the Indian subset of patients.

## 2. Material and Methods

The study was conducted in the Department of Gastroenterology, Maulana Azad Medical College and Associated G.B. Pant Hospital, New Delhi, which is one of the largest tertiary care centers located in north India, catering to a huge population from several neighboring states. It was a prospective observational analysis. The study was approved by the ethical committee of the institute. Informed consent was taken from all the patients and controls after the nature and possible consequences of the study were explained before enrollment in the study. A total of 257 patients were included in the study. One hundred thirty-one consecutive patients with diagnosed inflammatory bowel disease attending the outpatient department (opd) or admitted to the wards were enrolled, and a control group of one hundred twenty-six healthy controls was also included in the study. Most of the controls recruited were healthy relatives of IBS (irritable bowel syndrome) and IBD patients. Patients age 18 to 70 years old were included in the study. Patients with active tuberculosis, any history of diagnosed tuberculosis, and chest radiograph suggestive of old or active tuberculosis were excluded from the study.

Detailed history regarding the course and treatment of all the patients was recorded. History of flare-ups, extraintestinal manifestations, the presence of familial IBD, and smoking habits had been collected in a uniform format. Previous and concomitant medical therapy (steroid and/or immunosuppressive, or biological therapy) was registered. Clinical activity in CD was defined by Crohn's Disease Activity Index (CDAI) and in UC by a Clinical/partial Mayo Score (CMS). A patient was considered to be under an immunosuppressive therapy if any of the following criteria were fulfilled: intake of steroids for a period equal or superior to two weeks; intake of thiopurines, methotrexate, or cyclosporine for a period equal or superior to 2 months; prescription of anti-TNF-*α*; and intake of any two or three immunosuppressors associated with or in the absence of a concomitant anti-TNF-*α* prescription. Patients' medical history was screened for predefined TB risk factors including active malignant diseases, active hematological or oncological diseases, disease phenotype, duration of immunosuppressive therapy, number of immunosuppressive therapies, contact person in the daily life of the patient, workplace risk for infection, and previous TB infection. We also recorded the presence of a BCG scar as a sign of BCG vaccination.

Blood samples for IGRA were collected during routine laboratory testing on the same day when TST was performed. Prior to the tests, written informed consent was taken from all participants. Whole-blood interferon-*γ* (IFN-*γ*) release assay (QuantiFERON-TB Gold in tubes, Cellestis, Carnegie, Australia) based on specific peptides ESAT-6, CFP-10, and TB7.7 was performed and evaluated according to the manufacturer's recommendations [8, 9]. Standard TST was performed by Tuberculin PPD RT23 SSI (Staten Serum Institute, Denmark), which consists of an intradermal injection of tuberculin RT23 purified protein derivative (PPD) in 0.1 ml solution for injection into the inner surface of the forearm. A positive Mantoux reaction was defined with a predefined cut-off rate as a skin induration diameter at 72 hours after a skin test. A TST reaction of ≥10 mm of induration was considered positive. In special circumstances, a TST reaction of ≥5 mm of induration was considered positive like (a) patients with immunosuppression (including patients taking the equivalent of ≥15 mg/day of prednisone for 1 month or more or those taking TNF-*α* antagonists), (b) human immunodeficiency virus- (HIV-) infected persons, (c) recent contacts of a person with infectious TB disease, and (d) administration and measurement of the tuberculin skin test [5, 10].

### 2.1. Data Analysis

Categorical variables were described through absolute and relative frequencies, and continuous variables were described through the mean and standard deviation, or median and interquartile range (IQR), whenever appropriate. The prevalence of LTBIs was estimated with 95% confidence intervals (CIs) using QFT and TST. The concordance between these two tests was evaluated using the kappa (*κ*) statistic. The *κ* statistics were interpreted as follows: *κ* > 0.75 was considered to indicate excellent agreement, *κ* < 0.40 to indicate poor agreement, and *κ* between 0.40 and 0.75 to indicate fair to good agreement. Multivariate linear regression was used to compare groups to control confounding. To know the dependence, Pearson's correlation was calculated. All the reported *p* values were two-sided, and *p* values of <0.05 were considered statistically significant. The sample size was calculated using the latent TB prevalence of 40% with population correction, which showed the requirement of 120 subjects required in each group. All data were arranged, processed, and analyzed with SPSS v.23 (Statistical Package for Social Sciences).

## 3. Results

The study has recruited a total of 257 subjects including 131 IBD patients and 126 healthy controls.  Table 1 shows various demographic and clinical characteristics of the IBD patients. Among the IBD patients, most of the patients were in the age group of 18-40 years. The mean ages of the IBD patients and healthy controls included were 36.03 years (SD ± 12.5) and 46 years (SD ± 9.6), respectively. The majority of the study subjects were males, 65.1% in the IBD group and 55% in the control group. Out of the 131 IBD patients, 121 patients had ulcerative colitis and 10 patients had Crohn's disease. In the IBD patients, mean duration of illness of IBD was 46 ± 25 months. 23% in the IBD patient group and 31% in the control group had a BCG scar. Out of the 121 included UC patients, most patients (61.2%) had pancolitis (Montreal extent E3). According to the Clinical Mayo Score, at the time of evaluation, 31% of the ulcerative colitis patients had severe activity. Most of the parameters were comparable between the two groups (see Table 2); however, IBD patients had low hemoglobin and body mass index (BMI) as compared to control subjects.

### 3.1. Latent Tuberculosis Screening

**Figure 1:**
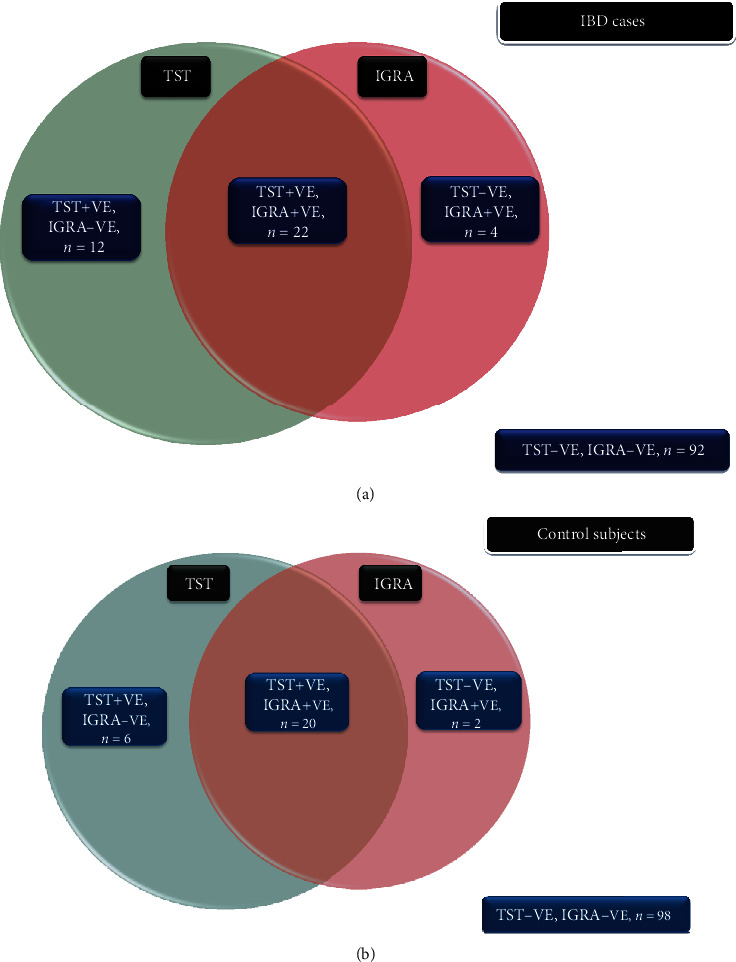
(a) Venn diagram shows the distribution of LTBI among IBD patients. (b) Venn diagram shows the distribution of LTBI among control subjects.

**Figure 2:**
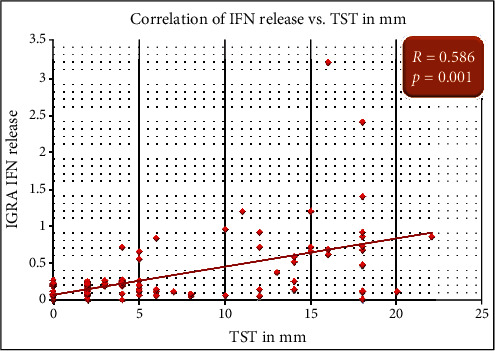
Correlation of IGRA: IFN release and TST measured in mm.

LTBI diagnosis was considered if any one of TST or IGRA was found to be positive. Out of a total of 257 participants, 66 (25.7%) were detected to have LTBI. 38 (29%) of the IBD patients and 28 (22%) of the control subjects had LTBI. The distribution of QFT-GIT and TST in IBD cases and controls is shown in Table 3 and Figures 1(a) and 1(b). Mean TST in IBD patients was 5.9 (±1.6); in IBD patients with positive TST, mean TST was -5.9 (±1.8), whereas it was 5.8 mm (±1.6) in control subjects (*p* value = NS) (Figure 2).

In BCG-vaccinated IBD patients, the TST and IGRA positivity rates were 22.4% and 18.7%, respectively, whereas the positivity rates were 19% and 16%, respectively, in healthy controls.

### 3.2. Agreement between TST and IGRA

**Figure 3:**
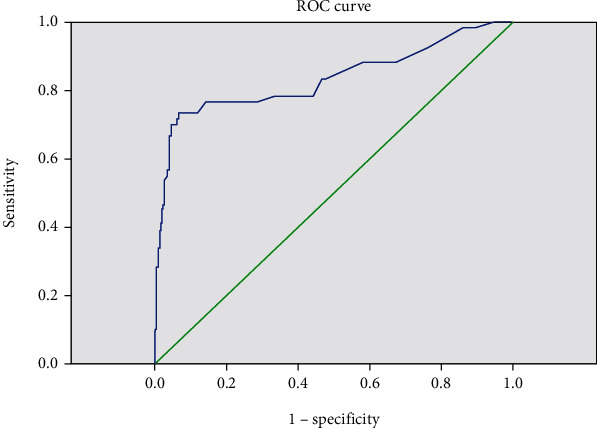
ROC curve for TST in mm with IGRA positivity.

We found that Cohen's kappa coefficient in IBD cases and control subjects was 0.66 and 0.79, respectively, which is indicative of a fair agreement (see Table 4 and Figure 3). Agreement test kappa value for IBD patients was 0.656 (CI 0.50-0.81), with a *p* value of <0.001. Analysis suggested that TST and IGRA were concordant tests. TST was found to be negatively correlated to BMI.  Table 5 shows logistic regression for factors affecting IGRA and TST positivity. Binary logistic regression showed that smoking and TST predict IGRA positivity.

**Figure 4:**
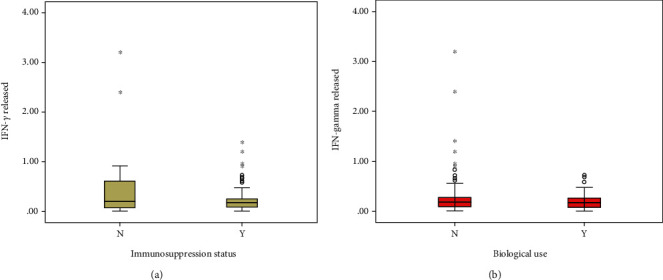
(a) Box plot comparing IFN-*γ* release according to immunosuppression. (b) Comparison of IFN-*γ* release according to biological use.

Effect of immunosuppressive agents and biological use on both TST and IGRA results were evaluated (see Tables 6(a) and 6(b)). There was no statistically significant difference concerning the mean TST values between the two groups. Quantitative IFN-*γ* release was assessed by quantity of IFN − *γ* test tube − nil tube and compared between two groups of IBD according to immunosuppression and biological use (Table 6). It was found that the mean IFN-*γ* released by the immunosuppressed group was significantly lower than that of the patients who were not immunosuppressed. We found that the mean IFN-*γ* released by a group of patients on biologicals was less than that of patients not on biologicals, although the difference was not statistically significant. Figures 4(a) and 4(b) show the comparison of IFN-*γ* release according to immunosuppression use and biological use, respectively.

## 4. Discussion

requent immunosuppressive treatment and malnutrition increase the chances of the reactivation of LTBI in patients with IBD. In particular, the use of TNF-*α* inhibitors and biologicals increases the risk of developing TB. Thus, screening for LTBI before the use of anti-TNF-*α* treatment is necessary since the reactivation of LTBI poses a potentially life-threatening complication [11]. Lacking a “gold standard” for the diagnosis of LTBI, TST and IGRA remain the primary screening tools despite having major limitations. An innovative IGRA and TST have been used in the surveillance of LTBI, but studies evaluating the comparative performance of TST and IGRA in patients with IBD are scarce and limited [12].

Detection and treatment of LTBI prior to anti-TNF therapy decrease the chance of active tuberculosis. Hence, it is equally important to evaluate the role of these diagnostic methods according to the prevalence of latent tuberculosis in a particular region or country, to guide the management of LTBI before immunosuppressive therapy. In our study, we compared these diagnostic methods in a large patient cohort and controls in a country with a high incidence of both latent and active tuberculosis. In our study, 257 subjects were analyzed including 131 IBD patients (121 were diagnosed with ulcerative colitis and 10 were diagnosed with Crohn's disease) and 126 healthy controls. Due to the lower prevalence rate of CD in India, our study had a lesser number of CD patients compared to other studies [13, 14]. As per the European Crohn's and Colitis Organisation (ECCO) guidelines for the management of opportunistic infections in IBD [11], LTBI diagnosis was considered if any one of TST or IGRA is found to be positive. According to these criteria, in our study, 66 (25.7%) of 257 participants were found to have LTBI. 38 (29%) of the 131 IBD patients and 28 (22%) among the 126 control subjects had LTBI. Studies by other authors have shown a prevalence rate between 15 and 24% [15].  Table 7 shows a comparison of various studies on the prevalence of LTBI in IBD patients.

No significant difference in LTBI was found between IBD cases and controls (*p* = 0.23). A previous study from India showed a prevalence of LTBI in 31% of healthy individuals. Joshi et al. showed that 51% of healthcare workers had LTBI, which could be explained by the higher exposure to infection in India [6, 7]. A study from South Korea which included only healthy adults, not an at-risk population, found only a 15% prevalence of LTBI [16].

In the current study, TST positivity was slightly higher than IGRA (23.3% vs. 19%). We found that the use of biologicals was associated with lower TST positivity (34% vs. 14%, *p* = 0.02); we did not find any significant difference in TST positivity among the immunosuppressed and non-immunosuppressed groups (*p* = 0.34). This may be explained by the defective T-cell function in patients on biologicals, a result that is supported by other studies with a similar conclusion [17, 18]. Our study demonstrated a fair agreement between TST and IGRA (kappa 0.656, 90.2% overall agreement). We found that agreement between both tests was similar between cases and controls and was not affected by immunosuppression status. The higher prevalence of LTBI may be the reason for the higher agreement between the two tests in our study. In our study, 23% in the IBD patient group and 31% in the control group had a BCG scar. BCG vaccination at birth may elicit a positive TST result in the absence of infection, and could therefore be responsible for a number of positive TST results. However, a relevant effect of BCG on TST positivity after a period of 15-20 years is less likely [19, 20]. In our study, we found that there was more TST positivity among BCG-vaccinated individuals (34% vs. 17%, *p* = 0.01); however, on logistic regression analysis, we did not find any association of BCG vaccination with TST or IGRA positivity. However, our study supports the superiority of IGRA over TST among BCG-vaccinated individuals. In our study, we found that 4% of participants had indeterminate QFT-GIT test results; it was higher among patients with biologicals (8%), although not statistically significant. This finding is also supported by Papay et al. [21].

Although immunosuppression was not found to affect overall LTBI prevalence, a major finding in our study is the negative impact of immunosuppressive therapy on the TST results and total IFN-*γ* released. Mean IFN-*γ* release was lower in the immunosuppressed group as compared to non-immunosuppressed individuals (0.26 ± 0.17 vs. 0.45 ± 0.07, *p* = 0.02). This is consistent with findings from a recent meta-analysis and other studies from Europe [21–24]. IGRA relies on the cell-mediated immunity to produce interferon-*γ* after stimulation with TB antigens; hence, any condition that alters the T-cell immunity may have an impact on the IGRA results. Immunosuppressive drugs may reduce the interferon-*γ* released by T cells, resulting in a reduced rate of positive result [28].

Our study is the first study from India to evaluate the prevalence of LTBI among IBD patients. Furthermore, the study included a well-matched control population to ascertain factors affecting the result of testing for LTBI. A well-characterized and diverse study population, with a fairly large sample size, and the measurement of TST and IGRA using a standardized technique are the strengths of our study. However, there are certain limitations in our study: (1) the study was not designed to follow the patients with latent TB for the development of active tuberculosis; [2] the inexistence of a gold standard reference test to diagnose LTBI, which would enable the identification of false positive and false negative results; and [3] we did not repeat the tests with negative results.

## 5. Conclusion

Our study is one of the few studies from a country like India where tuberculosis is highly prevalent that is aimed at evaluating the actual prevalence of LTBI among IBD patients and healthy controls. The study shows a prevalence of 25.7% of latent TB infection in IBD patients which is not significantly different from the control group. There was a fair agreement between TST and IGRA (91%, kappa value = 0.66). Although immunosuppression was not found to affect overall LTBI prevalence, it has a negative impact on the TST results and total IFN-*γ* released.

Screening of latent TB before the commencement of immunosuppressive therapy particularly in IBD patients pre-anti-TNF-*α* therapy is suggested. TST and IGRA tests are complementary methods.

## Figures and Tables

**Table 1:** Various demographic and clinical characteristics of IBD patients.

Patient characteristics	Ulcerative colitis	Crohn's disease
*n*	**121**	**10**
Male/female	**70/51**	**2/8**
Age (mean ± SD)	36.5 ± 12.6	29.8 ± 8.4
BMI (kg/m^2^) (mean ± SD)	22.2 ± 4.4	23.5 ± 2.8
Duration (months) mean ± SD)	46.6 ± 24	36.8 ± 16
Disease extent		
E1	**15 (12.4%)**	
E2	**32 (26.4%)**	
E3	**74 (61.2%)**	
Clinical Mayo Score (mean ± SD)	4.3 ± 2.5	
Disease location		
L1		**2**
L2		**1**
L3		**7**
L4		**—**
CDAI (med/range)		**276 (126-420)**
Immunosuppression	**89/121 (73.6%)**	**10/10**
Steroid	**89**	**2**
AZA	**56**	**10**
IFX	**38**	**6**
ADA	**1**	
Smoking	**37/121 (30.6%)**	**0**
BCG scar	**30/121 (25%)**	**0**
TST+ve	**33/121 (27.2%)**	**1/10**
IGRA+ve	**25/121 (20.7%)**	**1/10**
IGRA (test-nil) (mean ± SD)	0.30 ± 0.1	0.23 ± 0.1
TST MM (mean ± SD)	5.9 ± 3.1	4.1 ± 2.3

**Table 2:** Comparison between IBD group and control subjects.

	IBD patients (*n* = 131)	Control group (*n* = 126)	*p* value (IBD vs. control)
Age, mean (SD)	**36.0 (12.5)**	**45.9 (9.6)**	**0.001**
SEX (male) *n* (%)	**72 (55%)**	**82 (65%)**	NS
BMI (kg/m^2^) (SD)	**22.3 (4.3)**	**24.8 (3.4)**	**0.001**
BCG scar, *n* (%)	**30 (23%)**	**39 (31%)**	NS
Smoking, *n* (%)	**37 (28%)**	**30 (55%)**	NS
QFT test-nil (SD)	**0.3 (0.4)**	**0.3 (0.4)**	**0.06**
TST in mm (SD)	**5.9 (1.6)**	**5.8 (1.6)**	NS
HB (SD)	**10.5 (2.3)**	**14.4 (8.9)**	**0.001**
TLC (SD)	**8187.9 (2980.3)**	**8469.0 (1780.2)**	NS
Platelet (SD)	**280612 (114245)**	**275493 (114163)**	NS
Total protein gm/l (SD)	**6.8 (1.0)**	**7.6 (0.6)**	**0.001**
Albumin gm/l (SD)	**3.7 (2.7)**	**4.4 (0.4)**	**0.01**

**Table 3:** Distribution of QFT-GIT and TST in IBD cases and controls.

QFT-GIT in IBD cases and controls		Frequency	Percent	*p* value (*χ*^2^ test)
IBD patients	Positive	**26**	**19.8%**	**0.60**
	Negative	**99**	**75.6%**	
	Indeterminate	**6**	**4.6%**	
Control subjects	Positive	**22**	**17.5%**	
	Negative	**99**	**78.6%**	
	Indeterminate	**5**	**4%**	

TST in IBD cases and controls				
IBD patients	Positive	**34**	**26%**	**0.31**
	Negative	**97**	**74%**	
Control subjects	Positive	**26**	**20.6%**	
	Negative	**100**	**79.4%**	

**Table 4:** Pearson's correlation of TST and IGRA.

		QFT test-nil	TST (mm)	BMI (kg/m^2^)	Age	Albumin	HB
QFT test-nil	Pearson's correlation	—	**0.55**	-0.03	0.16	-0.05	0.15
	*p* value	—	**0.001**	0.71	0.08	0.58	0.09
TST in mm	Pearson's correlation	**0.55**	—	**-0.34**	-0.06	-0.06	0.13
	*p* value	**0.00**	—	**0.001**	0.51	0.49	0.13

**Table 5:** Logistic regression for factors affecting IGRA and TST positivity.

Logistic regression for factors affecting IGRA positivity				
	*p* value	ODD's ratio	95% C.I. for OR	
			Lower	Upper
Age (years)	0.63	1.02	0.93	1.12
Sex (male)	0.16	2.33	0.71	7.67
BCG vaccination	0.53	0.65	0.16	2.54
Smoker (yes)	0.001	0.06	0.01	0.34
BMI (kg/m^2^)	0.68	0.97	0.86	1.11
TST (mm)	0.001	0.68	0.61	0.75
Immunosuppression	0.94	0.92	0.11	7.72
Biological treatment	0.87	1.18	0.15	9.40

Age (yrs)	0.77	0.98	0.85	1.13
Sex (male)	0.40	0.48	0.09	2.67
BCG vaccination	0.08	7.56	0.82	70.14
Smoker (yes)	0.19	4.59	0.47	45.11
BMI (kg/m^2^)	0.02	0.78	0.63	0.95
Immunosuppression	0.46	4.69	0.08	2.55
Biological treatment	0.21	12.41	0.25	6.20

**Table:** (a) Comparison of TST according to immunosuppression and biological use

TST according to immunosuppression				
	Immunosuppression	*n*	Mean ± SD (mm)	*p* value (independent sample unpaired *t*-test)
TST	Yes	**99**	5.8 ± 1.34	**0.08**
	No	**32**	5.8 ± 1.5	
IFN-*γ* release according to biological use				
TST	Yes	**38**	5.6 ± 1.2	**0.07**
	No	**93**	5.8 ± 1.4	

**Table:** (b) Comparison of IFN-*γ* release according to immunosuppression and biological use

IFN-*γ* release according to immunosuppression				
	Immunosuppression	*n*	Mean ± SD	*p* value (independent sample unpaired *t*-test)
QFT-GIT (test-nil tube)	Yes	**99**	0.26 ± 0.17	**0.02**
	No	**32**	0.45 ± 0.07	
IFN-*γ* release according to biological use				
QFT-GIT (test-nil tube)	Yes	**38**	0.23 ± 0.13	**0.06**
	No	**93**	0.34 ± 0.07	

**Table 7:** Comparison of various studies upon the prevalence of LTBI in IBD patients.

Study	Country	No. of patients (*n*)	Control (*n*)	Male (%)	Mean age (yrs)	IBD UC/CD	BCG (%)	TST+ve (%)	IGRA +ve (%)	IGRA method	Indeterminant IGRA (%)	Kappa (*κ*) concordance/agreement TST/IGRA
Papay et al., 2011 [21]	Austria	208	—	49	36	152/56	—	12.5	7.2	QFT-GIT	7.7	0.21
Schoepfer et al., 2008 [12]	Switzerland	212	44	49	31	44/114	70	18	8.3	QFT-GIT	3	0.029
Andrisani et al., 2013 [15]	Italy	92	50	39.6		60/32	—	15.2	14.1	QFT-TST	0.9	0.51
Arias-Guillen et al., 2014 [25]	Spain	205	—	50	44	157/48	—	26.8	7.8	QFT-GIT, TSPOT	8.4	0.34
Wong et al., 2014 [20]	China	268	234	160	43	128/136	95	9.9	20.6	QFT-GIT	4	0.19
Kurti et al., 2015 [26]	Hungary	166	—	54	24	122/44	—	21	8.4	QFT-GIT	0.6	0.41
Abreu et al., 2016 [27]	Portugal	250	—	44	36	81/19	—	23	10	QFT-GIT	2.2	0.22
Our study	India	**131** *IBD*	**126**	**60**	**40.1**	**121/10**	**26**	**23.3**	**18.9**	*QFT-GIT*	**4.3**	**0.66**

## Data Availability

All the necessary data are included in the manuscript.
